# Smoke Emission Properties of Floor Covering Materials of Furnished Apartments in a Building

**DOI:** 10.3390/ijerph17239019

**Published:** 2020-12-03

**Authors:** Marzena Półka, Anna Szajewska

**Affiliations:** The Main School of Fire Service, 01-629 Warsaw, Poland; aszajewska@sgsp.edu.pl

**Keywords:** smoke emission, fire environment, visibility in smoke, floor covering

## Abstract

The paper presents results of tests related to smoke optical density conducted on four various textile floor coverings for the needs of building interior design. Smoke emission is one of basic elements that characterize the fire environment. Consequently, the objective of the paper was to carry out a comparative analysis of smoke generation of chosen floor coverings for selected thermal exposures and in the presence or absence of a stimulus igniting the volatile gaseous phase (pilot flame). For the needs of our experimental research use was made of polypropylene, polyester, composite of wool, cotton, viscose and polyamide floor coverings. The highest value of the maximum specific optical density of smoke (494.7) was recorded for the floor covering consisting of 100% polypropylene (with higher fiber) under flameless combustion conditions (without the pilot flame). The polypropylene floor covering without underlay proved to be the best material from among all the tested ones with respect to smoke generating properties, and its samples offered the lowest value of optical density after 4 min for testing variants without the application of a pilot burner, with the flammable phase of decomposition products of this sample during the testing in which the burner was used to ignite at the latest. Experimental research has been carried out based on the standard ISO 5659–2:2017–08. The tests results were compared with international optical smoke density requirements for the interior design of ships and trains.

## 1. Introduction

Problems related to fire hazards and the consequent fire safety of textile materials are truly important, because as may be seen from statistical data published in the Information Bulletin of the National Headquarters of the State Fire Service in Poland for 2018, almost 22.43% of all fires (to be precise 33,522 fires) took place in residential buildings [1]. Smoke as a basic heat conveyor in a fire poses a major threat for people’s health and environmental because of the inhalation of toxic substances which are integral part of it and attenuation of fire spaces and neighboring enclosures. As regards safety, textile or polymeric floor coverings are flammable materials, and their ignitability and smoke generation depend to a large extent on their chemical composition, density and thickness of the element.

Used for finishing and fit-out of interiors, textile and upholstered materials tend to occupy large areas in buildings, and hence should display an appropriate degree of flammability, which depends on the functionality of those materials. Carpeting consists of pile, which is simply a top covering layer, and the base on which it is set. Depending on the manufacturer’s preferences, the carpet pile may come in different shapes, such as for example loops, dense and rigid fibers or tangled fibers. In order to allow the determination of fire hazards of diverse textiles, including carpeting, it is necessary to identify fire hazards such as combustion rate, flame spreading ratio, values of combustion heat, characteristic features of the qualitative and quantitative composition of the decomposition and combustion products and smoke emission. Many different research centers have taken up issues related to the flammability of textile-based carpeting or fibers used in textile products [2,3,4,5]. A review of available references shows that given the growing needs and expectations of the consumers and the latest technical achievements and technological innovations, carpeting producers are developing innovative combinations of chemical compounds, which determine properties such as color, structure, unit weight or durability [6,7]. However, the most common raw materials used for carpet pile include polypropylene, polyamide or wool fibers. Quite frequently use is also made of mixtures of those substances. As regards natural fibers, wool is characterized by limited flammability, and as a rule wool-based materials have a relatively high value of the oxygen ratio of ca. 25% by volume and low flame temperature of ca. 680 °C [5]. The natural capability of fire resistance offered by wool fibers arises from the chemical composition and formation of a carbonized coat on the surface of materials, forming in such a way a barrier for heat and oxygen, and as effect delaying combustion. Agents that reduce flammability and at the same time facilitate carbonization of wool include compounds of boric acid/mixtures of borax, salts of sulfonic acid and ammonium salts, such as ammonium phosphates and sulfates. The most commonly used and lasting modification process of wool and wool products is the Zirpro process [2]. An advantage of this process is the lack of any discoloration or other influence on the aesthetic values of wool. Synthetic fibers made of semi-crystalline polyamides (PA) are also used for the production of textile carpeting. They are commonly used in textiles similarly to polyesters, despite certain difficulties connected with modifications of those compounds with the aim of assuring lasting combustion retardation thanks to in inclusion of additives. This difficulty arises from their reactivity in the melted state, and recent discoveries on the scope of fire resistance of those compounds pertain primarily to the introduction of nanoparticles into the polymer base [3]. Other fibers used for carpeting are polypropylene fibers (PP) which offer a high tensile strength and are relatively cheap. PP fibers are also commonly used for the production of clothing, upholstery, in the automotive industry, car upholstery, in diverse household textiles, wall coverings etc. Due to the fully aliphatic structure of this hydrocarbon, polypropylene burns in the flame very quickly, without generating smoke or carbonized remnants. The team of Zhang and Horrocks [8] described various approaches for the manufacturing fire resistant fibers made of PP. A perfect approach to the modification of polypropylene fibers is based on introducing of a flammability reducing agent into the fiber. Apart from the abovementioned raw materials, carpeting is also produced using several other chemical substances aimed at improving aesthetic values, durability of the material or its physical and chemical properties. They include inter alia adhesives for binding fibers, dye solvents, antistatic substances and compounds preventing excessive material staining.

Not only the flammability of textile carpeting needs to be studied, but also the smoke generating properties must be analyzed. This is due to the fact that statistical data show that the direct cause of more than 80% of all fatalities which happen during fires are hazards arising from the presence of smoke [9]. It should be borne in mind that restricted visibility hinders or obstructs the evacuation process of people who need to exit the building. In many cases if the visibility range becomes reduced as a result of smoke logging, this becomes the principal risk factor for people inside a building compromised by a fire, before its thermal impact may be initiated [10]. Minimizing the fear caused by an imminent hazard contributes to better efficiency of the human body under fire conditions, which in turn helps control panic and facilitates well-organized successful rescues and fire suppression activities. A particularly intensified smoke emission occurs in conditions where there is a shortage of oxygen. As an effect combustion may be incomplete or partial, and products created due to such transformations form the chief elements of the smoke in the dispersed phase. There are several factors that affect the smoke emission properties during the combustion process of floor panels, and they range basically from the specific kind of material (chemical composition) to the actual type of combustion. Evacuation from buildings and extinguishing of the fire may be seriously impeded by dense smoke that may be created by burning materials. The feeling of impending danger may be significantly increased by limited movement possibilities on an area comprised by a fire, and in addition the body capacity may be impaired and uncontrolled panic ensues [11,12,13] It is generally recognized that particular hazards may arise in places having a limited height and cubage, as they become filled with smoke relatively quickly, causing a direct danger to people present inside them. The most distinctive trait of a fire is the generation of smoke, which is a phenomenon that seriously impedes rescue and extinguishing actions. When intense dense smoke is generated during the combustion process, rescuers have difficulties with executing their activities as it is not easy to gain access to the place of origin of a fire; also victims in need of evacuation are affected by it and what is more, it leads to changes in the surrounding environment [14].

When selecting materials to be used as interior finishing elements in buildings, first of all aesthetic values, resistance to wear and tear and comfort of usage are taken into consideration. In most cases elements used for fit-out of building interiors are principally made of plastics, a type of material that at elevated temperatures very easily undergoes thermal decomposition processes and at the same time generate fairly large amounts of smoke [15,16]. The scale of this hazard keeps escalating owing to the continuously growing usage of polymer materials in the construction industry, because these materials make up about 10–15% of the total mass of flammable materials that are used in apartments. The present paper undertakes to determine the smoke generating properties of textile floor covering applied in interior fit-outs and a reference is made to results covered by maritime and railway standards.

## 2. Materials and Methods

For the needs of our experimental research use was made of samples of five different floor coverings. Table 1 presents the characteristics of the composition of the samples of floor covering used for the studies. The floor covering types used in testing were representative commercial products that are most commonly used for interior decoration (Table S1). There were certain differences between the used samples, such as chemical composition or, in the case of PP floor covering, the fiber height and presence or absence of a base layer. The average thickness of the underlay ranged between 6 and 7 mm. Given its relatively simple production technology, polypropylene floor covering is a standard and relatively cheap commercial product. It was found that sample No. 5 had the highest fiber height. The tested floor coverings did not contain flame retardants.

According to the recommendations specified in the standard EN ISO 5659-2 [17], testing was conducted with the use of 75 × 75 mm samples with a maximum thickness of 25 mm, which had been wrapped in aluminum foil and placed in a special grip furnished with a washer made of fire resistant fibers to insulate the bottom part of the sample. The smaller thickness of the floor covering samples was compensated by providing more insulating fabric under the samples to assure a 25 mm distance between the lower part of the radiator and the exposed surface of the sample. Thickness of the tested sample was up to 20 mm. Experimental research has been carried out based on the standard EN ISO 5659-2 [17].

### Characteristic Features of The Research Method

To determine the smoke generating properties of the selected materials, the so-called single-chamber test was performed according to EN ISO 5659-2 [17]. In this test method a material sample prepared in line with the requirements of the standard [17] is placed in the chamber, the surface of which is exposed to external density heat flux (from an electric cone radiator) amounting up to 50 kW/m^2^ or less, depending on the type of test. Smoke generated as an effect of thermal decomposition of the sample is collected in a closed chamber, inside which an optical-measuring device transmits a light beam concurrently measuring the T_ro_—initial transmittance of light [%] and T_r_—light transmittance [%] in the function of the sample burning time.

The basic characteristic feature delimited on the basis of conducted measurements was a change of specific optical density of smoke in the time function, determined in accordance with the Equation (1) [17]. A single measurement was continued for 20 min:

(1) $$D_{s}=\frac{V_{k}}{A\cdot L}\log\frac{T_{\mathrm{ro}}}{T_{r}}=\frac{V_{k}\cdot D}{A\cdot L}$$

where D_s_—specific optical smoke density, V_k_—volume of measurement smoke chamber [m^3^], L—thickness of smoke layer [m], A—active surface of sample [m^2^], T_ro_—initial transmittance of light [%], T_r_—light transmittance [%] and D—optical density of smoke.

The specific optical density of smoke D_s_ is understood as the optical density of a 1 m thick smoke layer produced in a volume of 1 m^3^ during combustion or thermal decomposition of tested material per area unit of materials equaling to 1 m^2^.

To allow executing a wider analysis, derivative values such as the time until achievement of D_s_max_, and the value of specific optical density after 4 min D_s_4_ and area under curve of specific optical density during the first 4 min V_OF4_. were also recorded. Once the maximum value of D_s_max_ has been achieved, the value of specific optical density decreased. This took place as a result of vanishing of smoke due to coagulation, sedimentation, evaporation and condensation on the chamber walls [18].

In Poland the criteria for the selection of materials are specified by relevant regulations, such as for example the Regulation of the Minister of Infrastructure on technical criteria to be met by buildings and their location or for interior fittings and finishing applicable to all materials used in all types of construction works, standard EN 45545-2 applicable to materials used in the railway industry [19], and the International Code for Application of Fire Test Procedures (FTP Code) applicable to shipbuilding [20]. Unfortunately, those minimum criteria do not always assure the required safety in the case of fire. It should be borne in mind that requirements valid for interior materials may not be applied in residential buildings. Meanwhile according to fire statistics fires tend to occur generally in residential buildings and transport. For example, it is not permissible for floor coverings used in interior fittings in ships to have specific optical density higher than 500. The D_s_max_ values were used to determine requirements concerning smoke generating properties of materials and components used in railway industry according to standard EN 45545-2, and smoke generation of materials and products were determined by parameters given in standard [17]. Measurements method is applied in maritime construction (International Code Part 2) and in railway construction, and the scope of parameters being delimited has been determined depending on the designation of test results. In the railway construction use is made of standard [19], which defines requirements with respect to smoke generation of materials used in railway vehicles, classified according to operating and design categories, designated for transporting passengers on European railway lines.

The following parameters are used for classification of materials:

- D_s_max_—maximum specific optical density of smoke achieved during testing,
- D_s_4_—specific optical density of smoke after a time of 4 min,
- V_OF4_—area under curve of specific optical density during the first 4 min determined in accordance with the Equation (2) [17]:

(2) $$V_{\mathrm{OF}4}=\int_{0}^{4}D_{s}\cdot \mathrm{dt}$$

**Figure 1:**
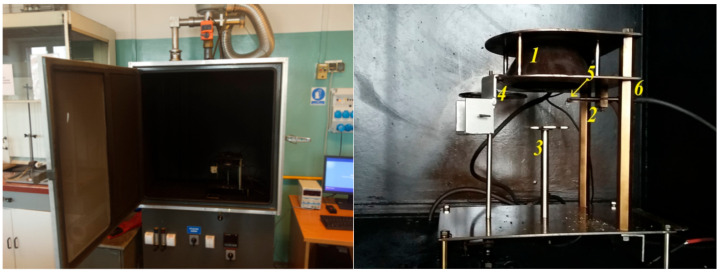
View of chamber and cone radiator for testing optical density of smoke: 1. Cone radiator, 2. Pilot burner, 3. Base for fixing the sample, 4. Shutter, 5. Sparker, 6. Gas valve.

Two configurations were adopted for the needs of the tests, namely the use of a piloted flame to ignite thermal decomposition products and without it. The value of external density radiation heat flux was adopted at the level of 25 kW/m^2^ (with or without piloted flame) and 50 kW/m^2^ (without piloted flame) according to the standard measurement conditions [19]. Figure 1 shows the research smoke chamber. The value of the external heat flux applied to the surface of tested samples was selected according to requirements of standard and testing conditions applicable for the materials to be used as lining on ships or in railways. One or two ignition sources were applied in the tests, one of them was an electric radiator used in each test to obtain the chosen external heat of flux—the other was a piloted flame. It means that tested materials were burnt in an external heat of flux with or without of piloted flame.

## 3. Results and Discussion

Average values of three measurements of defined parameters are presented in Table 2, Table 3 and Table 4. 

The amount of smoke released from the tested materials depends not only on the value of the thermal flux, but also on the presence of a pilot flame that burns the combustible gas phase, and in this case we observe a lower value of D_s_max_. During combustion at 25 kW/m^2^ without the piloted flame, all tested samples reached the maximum value, i.e., D_s_max_ 494.7 for sample No. 5 containing 100% of polypropylene, while during combustion with piloted flame at 25 kW/m^2^ the lowest D_s_max_ value was reached by a floor for No. 1 sample (80% wool, 16% cotton, 3% viscose, 1% polyamide)—85.9 and it was lower by 325% than the value obtained for the No. 3 sample (100% polyester), which D_s_ max_ in these test conditions was 365.4. 

Sample No. 4 (100% polypropylene floor covering without underlay) proved to be the best material from all the tested ones with respect to smoke generating properties, and its samples have offered the lowest value of specific optical density after 4 min for testing variants without the application of the piloted flame, with the flammable phase of decomposition products of this sample during the testing in which the burner was used to ignite at latest. 

Sample No.1 (80% wool, 16% cotton, 3% viscose, 1% polyamide) exerts the most advantageous impact on the value of specific density and the ignition time of flammable gaseous phase, while material No. 3 sample (100% polyester) achieved the highest D_s_max_ values during testing in the presence of the piloted flame. Polyester contains a relatively high number of oxygen atoms as compared to wooden and PP floor coverings (which lack oxygen atoms), and this causes ignitability of their thermal decomposition product from a small flame and as a result increases the value of the specific smoke density.

Those higher values obtained during the decomposition of samples of floor covering could be caused by the fact that apart from fiberboard their structure comprises other layers that are bound by glue. This could cause an increase in the D_s_max_ value.

D_s_max_ values for sample No. 5 during exposure to radiation with the value of 25 kW/m^2^ (without the presence of the burner) were clearly the highest from among the studied materials (higher than floor covering No. 1 (with external flame) by 476%, and this was the biggest difference).

A review of data allows the presumption that floor covering underlay contributed to a large extent to such a high D_s_max_ value. An insulating underlay would help achieve a smaller heat loss out the back of the sample leading to higher temperatures and higher heat production and consistently tested floor covering contained underlay have producing more smoke and showed the biggest values of specified optical density than tested floor covering without of underlay, respectively to the applied heat exposure and present piloted flame. Two measurement samples no 1 and 4 containing no underlays were found to have a lower D_s_max_ (sometimes even more than twice at 25 kW/m^2^ without piloted flame).

Literature sources [22] indicate that PP floor coverings/carpets unmodified by flame retardants are also characterized by high heat release rate dynamics. The HRR max values obtained using the cone calorimeter were: for PP floor coverings 396 kW/m^2^, for 100% wool carpets 287 kW/m^2^, for carpets 80% wool 20% PA 385 kW/m^2^, 100% PA-174 kW/m^2^ at external heat flux of 50 kW/m^2^. According to [22] the highest combustion heat values were recorded for PP floor coverings 35.232 kJ/g, for 100% wool carpets 19.041 kJ/g, for 80% wool carpets 20% PA 19.873 kJ/g, 100% PA 21.828 kJ/g.

This arises from the fact that contrary to polymeric fibers thermal decomposition products it has a higher value of maximum specified optical density during flameless combustion than flame in conditions. Consequently, it is clear that the smoke release intensity is of great impact for limiting the visibility splay in smoke. The visibility range is considered as the biggest distance in smoke from which a given object is visible [23]. It is a function of optical properties of smoke, type of light transmitted by the given object (emitted or reflected), type of lighting of the premise by external light sources and initial contrast of the object [23,24,25,26,27]. Experimental works of Jin, Rasbash et al. allowed finding a relation between the extinction coefficient $K_{s}$ and the visibility range W in smoke [23,28,29]:

(3) $$W=\frac{C}{K_{s}}$$

where W—visibility range indicates the biggest distance from which the observed item is visible in a smoke logged compartment [m], C—dimensionless constant that characterizes illumination of the monitored item in smoke (shining with its own light-emitted or with reflected light) with values of C_emited_ = 8.0 and C_reflected_ = 3.0 respectively pursuant to [19].

Taking into account dependence (3) and Equations (4) and (5) and assuming as D_s_ its maximum value D_s_max_ obtained was dependence (6) on the maximum value of visibility range ($W_{\max})$:

(4) $$\frac{D}{l}=\frac{K_{s}}{2303}$$

(5) $$\frac{D}{l}=D_{s}\cdot \frac{A}{V_{k}}$$

where V_k_–volume of the research chamber 0.75 [m^3^], A–active surface of sample 0.0042 [m^2^] and D is the optical density of smoke

For the tested carpeting burnt in the flameless way at an exposure of 25 kW/m^2^ maximum visibility ranges ($W_{\max})$ have been calculated according to Equation (6):

(6) $$W_{\max}=\;\frac{C\cdot V_{k}}{2303\cdot D_{s\_\max}\cdot A}$$

**Figure 2:**
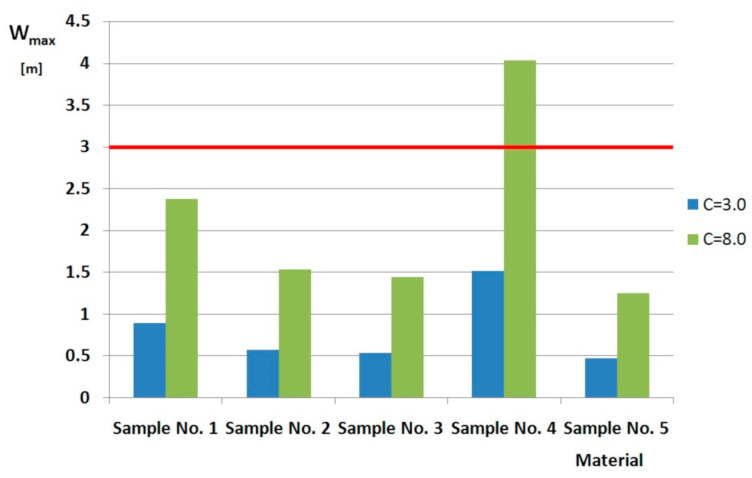
Diagram of the maximum visibility for tested materials.

A collective listing of the values of the maximum visibility ranges in smoke for the tested materials is presented in Figure 2. The maximum visibility values in smoke of the tested floor covering depend on their chemical composition and structure of the product. Taking into consideration the same chemical composition it may be presumed that the presence of an underlay and a bigger height of the PP carpeting fiber (sample 5) as compared to sample 4 determines a higher value of both D_s_max_ as well as the lowest value of the visibility range in smoke in the analyzed combustion conditions. The PP floor covering (sample 4) as compared to other tested floor coverings was found to have the biggest visibility parameters both for C-3.0 (fluorescent signs, light reflecting signs) and C-8.0 (illuminated by their own light).Consequently the tested PP floor covering that undergoes flameless combustion causes a bigger limitation of visibility, which is an effect of more intense smoke generation. The smallest visibility range (below 0.5 m) during the testing was achieved for carpeting No. 5.

The biggest visibility range in smoke equaling to 4 m was obtained for sample No. 4 in the analyzed flameless combustion. The composition of this sample comprised 100% PE, and the smallest height of fibers has been recorded as has already been found before—leading to a reduction of smoke generation of the material, and hence appropriate enhancement of visibility in smoke.

Visibility in smoke allows moving inside a building during a fire. It is assumed that the minimum visibility range in smoke that allows safe evacuation is 3–5 m for a familiar building and 15–20 m for an unknown building [12]. This allows the presumption that if the premise selected for the calculations were in a building known to people being evacuated and a fire broke out in it, the amount of smoke generated from decomposition (in conditions of flameless combustion) only carpeting No. 4 could allow safe evacuation of people from the building, because the visibility range calculated for it equaled to more than 3 m. On the other hand, safe evacuation cold not have been executed for all the tested carpeting types in conditions of their flameless combustion in an unknown building, because the visibility range set out for them was below 15 m.

## 4. Conclusions

The following conclusions may be drawn from results of conducted studies and from a review of data obtained from literature:

- Limitation of visibility by generated smoke depends not only on the thermal flux, but also on the presence of flame during combustion, which ignites the gaseous phase, and as a rule in such conditions the value of D_s_max_ becomes reduced.
- At the thermal flux of 25 kW/m^2^, without the pilot flame, floor covering No. 5 (100% PP with underlay and 0.7 mm fiber) was found to produce the highest values of the maximum specific optical density of smoke (494.7). Nevertheless, it needed the shortest period of time to achieve the maximum specific optical density of smoke. It required 465 s less than the longest time for those combustion conditions. For this reason, it is considered to be the most dangerous of all the tested carpeting types, given the criterion of specific optical density and the time of smoke generation in conditions of flameless combustion. On the other hand, the lowest of the maximum specific optical density of smoke was recorded for carpeting No. 4 (100% polypropylene, without undelay and with 0.3 mm fiber). The time needed to achieve the maximum upper threshold of specific optical density of smoke was the second as to the value as compared to the remaining materials (100 s fewer than the longest time), which proves high security values of this type of carpeting.
- At the thermal flux of 25 kW/m^2^, at the presence of a pilot flame, values of the maximum specific optical density were clearly lower. The highest value of the maximum specific optical density of smoke was recorded for floor covering No. 3 (100% polyester, with underlay, and 1.5 mm fiber) (365.4). The time until achievement of this value was by 102.5 s shorter than the longest time from all the tested materials. The lowest value of the maximum specified optical density of smoke was recorded for carpeting No. 1 (80% wool, 16% cotton, 3% viscose, 1% polyamide). Furthermore, the time needed to achieve this threshold was the longest one from the studied carpeting types in those specific conditions, which additionally enhances safety aspects in case of potential ignition of this material.
- At the thermal flux of 50 kW/m^2^, without the pilot flame, the highest value of the maximum specific optical density smoke of was once again recorded for 100% polyester carpeting (418.6). Similarly, like during the trial with the present pilot flame, the time needed to achieve the maximum specific optical density of smoke was lower by 87.5 s than the longest time from among the tested materials. Two samples have achieved a similar value of specific optical density, which concurrently was the lowest one: carpeting No. 4 (100% polypropylene, without undelay and with 0.3 mm fiber) and No. 1 (80% wool, 16% cotton, 3% viscose, 1% polyamide). Given the much longer time (a difference of 100 s) needed to achieve the maximum value of specific optical density of smoke, carpeting No. 4 has proven to be the most dangerous product taking into account parameters and time until smoke generation.
- Floor covering 100% polyester has twice the achieved the highest maximum specified optical density of smoke. This material is almost fully made of polyester. Unfortunately, none of the remaining samples subjected to testing comprised this compound in its composition, which renders impossible the determination of the extent of its impact on the value of the maximum specific optical density of smoke.
- Floor covering No. 5 (100% PP with underlay and 0.7 mm fiber) has twice proven to be the most dangerous material in no-flaming combustion condition given its properties and the time necessary to generate smoke. It almost fully consists of polypropylene (also carpeting No. 2 and 4 have the same composition).
- The biggest visibility range in smoke equaling to 4 m was achieved also for sample No. 4 in the analyzed flameless combustion. The composition of this sample comprised 100% PE, but this carpeting had the lowest number of fibers as has already been ascertained before—a considerable reduction of smoke generation properties of the material, and consequently also improved visibility range in smoke.
- Pursuant the FTP Code to, Part 2:2010 “The material meets maritime requirements with respect to smoke generation when mean values of the maximum specific optical density D_s_max_ for none of requirements pertaining to thermal decomposition and combustion conditions exceed the following admissible values: i.e., D_s_max_ ≤ 500 for floor covering – the average optical density of none of the tested research materials has exceeded the specified threshold value, which confirms its consistence with maritime requirements contained in the International Code for the Application of Fire Test Procedures.
- In conditions that prevail during an actual fire quick smoke release constitutes a significant hazard for people due to the practically immediate limitation of visibility and direct exposure to high concentrations of toxic gases. Limiting of the visibility range in smoke is the main hazard to humans during a fire, which hinders or even completely impedes the safe escape of people from a building. Limiting the visibility range in smoke depends on the capacity of the premises, for which it is being set out, chemical composition of the material burning inside the premise, rate and amount of smoke generated from the material and illumination of escape signs provided in a smoke-logged premise.

The conducted experimental study confirms the thesis that the smoke-generating properties of materials—in this case floor coverings—are determined not only by their chemical composition (type of polymer, material and their proportions), but also the type and conditions of their combustion (flame combustion, flameless combustion). The combustion of tested materials in conditions of an actual fire would consequently lead to quick smoke-logging of premises and hindered evacuation of the people.

## Figures and Tables

**Table 1:** Identification and composition of the tested samples.

Sample Determination	Composition (%)	Fiber Height (cm)	The Present of Underlay in Floor Covering
Sample No. 1	Wool 80 Cotton 16 Viscose 3 Polyamide 1	0.5	none
Sample No. 2	Polypropylene 100	0.6	yes
Sample No. 3	Polyester 100	1.5	yes
Sample No. 4	Polypropylene 100	0.3	none
Sample No. 5	Polypropylene 100	0.7	yes

**Table 2:** Listing of average values of delimited parameters—tests at external heat flux 25 kW/m^2^ without the presence of external piloted flame [21].

Name of Sample	Average Mass (g)	Average Time to the Ignition (s)	Average D_s_max_	Average Time Until Achievement of D_s_max_ (s)	Average D_s_4_	Average V_OF4_ (min)
Sample No. 1	6.8	no ignition	260.7	852.5	138.6	256.3
Sample No. 2	6.9	no ignition	403.1	1005.0	246.3	404.4
Sample No. 3	13.3	no ignition	430.6	1187.5	56.8	56.8
Sample No. 4	1.7	no ignition	153.6	1087.5	37.7	63.4
Sample No. 5	7.9	no ignition	494.7	722.5	316.2	536.0

**Table 3:** Listing of average values of delimited parameters—tests at external heat flux 25 kW/m^2^ with the presence of external piloted flame [21].

Name of Sample	Average Mass (g)	Average Time to the Ignition (s)	Average D_s_max_ (-)	Average Time Until Achievement of D_s_max_ (s)	Average D_s_4_ (-)	Average V_OF4_ (min)
Sample No. 1	6.2	276.5	85.9	352.5	75.0	150.6
Sample No. 2	7.2	471.5	230.6	157.5	205.1	516.4
Sample No. 3	13.5	488.5	365.4	250	342.3	544.9
Sample No. 4	1.5	95.5	129.6	90	106.5	340.5
Sample No. 5	8.0	460	158.3	135	138.8	391.7

**Table 4:** Listing of average values of delimited parameters—tests at external heat flux 50 kW/m^2^ without the presence of external piloted flame [21].

Name of Sample	Average Mass (g)	Average Time to the Ignition (s)	Average D_s_max_ (-)	Average Time Until Achievement of D_s_max_ (s)	AverageD_s_4_ (-)	Average V_OF4_ (min)
Sample No. 1	6.7	384.5	115.7	232.5	110.1	334.8
Sample No. 2	6.8	486.5	236.2	195	230.9	684.1
Sample No. 3	13.4	615	418.6	245	394.4	1079.4
Sample No. 4	1.7	148.5	115.3	332.5	93.0	327.9
Sample No. 5	8.0	474	286.3	142.5	275.5	844.9

## Supplementary Materials

The following are available online at https://www.mdpi.com/1660-4601/17/23/9019/s1, Table S1: Photos of the tested samples.
