# Supplementary figures and images for: Smoke Emission Properties of Floor Covering Materials of Furnished Apartments in a Building
Source: Int J Environ Res Public Health. 2020 Dec 3;17(23):9019. doi: 10.3390/ijerph17239019 (PMC7730770; doi:10.3390/ijerph17239019)

Supplementary material

Table 1. Photos of the tested samples.

| Number samples | Photo                                                                                |
|----------------|--------------------------------------------------------------------------------------|
| 1              | 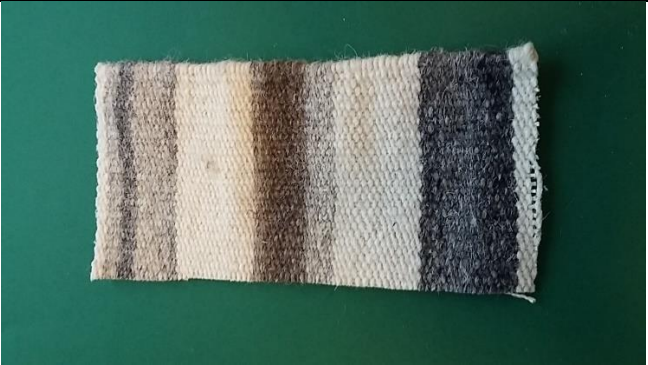   |
| 2              | 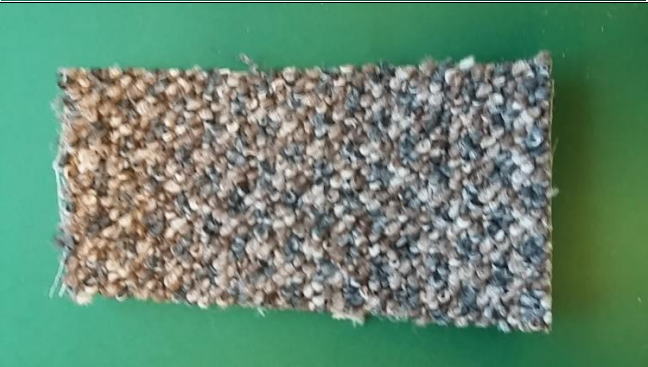  |
| 3              | 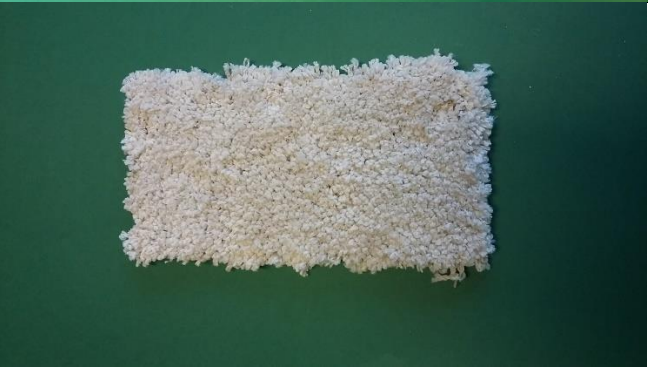 |
| 4              | 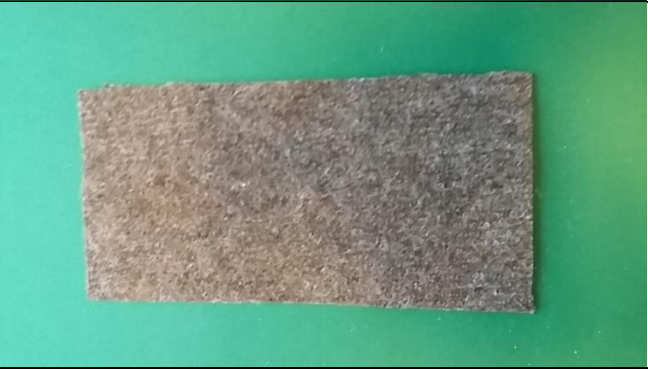 |

5

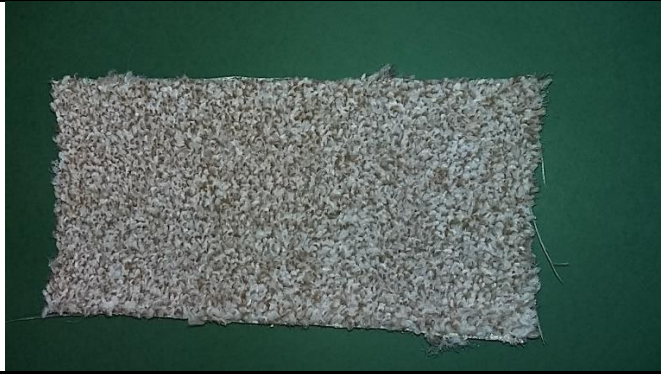

Supplement: Supplementary file 1 [file ijerph-17-09019-s001.pdf]
